# Circulating tumor DNA laboratory processes and clinical applications in nasopharyngeal carcinoma

**DOI:** 10.3389/fonc.2025.1520733

**Published:** 2025-05-15

**Authors:** Ziman Wu, Haiyan Yang, Xinying Li, Xiang Ji, Chan Mo, Zhou Zheng, Yafei Xu, Dan Xiong

**Affiliations:** ^1^ School of Medical Technology, Xinxiang Medical University, Xinxiang, China; ^2^ Medical Laboratory of the Third Affiliated Hospital of Shenzhen University, Shenzhen, China; ^3^ Shantou University Medical College, Shantou, China; ^4^ Department of Cell Biology and Genetics, Shenzhen University Health Science Center, Shenzhen, China

**Keywords:** CtDNA, pre-analytical, detected methods, biomarker, nasopharyngeal carcinoma

## Abstract

Circulating tumor DNA (ctDNA), a subset of cell-free DNA (cfDNA), originates from primary tumors and metastatic lesions in cancer patients, often carrying genomic variations identical to those of the primary tumor. ctDNA analysis via liquid biopsy has proven to be a valuable biomarker for early cancer detection, minimal residual disease (MRD) assessment, monitoring tumor recurrence, and evaluating treatment efficacy. However, despite advancements in ctDNA analysis technologies, standardized protocols for its extraction and detection have yet to be established. Each step of the process—from pre-analytical variables to detection techniques—significantly impacts the accuracy and reliability of ctDNA analysis. This review examines recent developments in ctDNA detection methods, focusing on pre-analytical factors such as specimen types, collection tubes, centrifugation protocols, and storage conditions, alongside high-throughput and ultra-sensitive detection technologies. It also briefly discusses the clinical potential of liquid biopsy in nasopharyngeal carcinoma (NPC).

## 1 Introduction

First described by Mandel and Metais in 1948, circulating cell-free DNA (cfDNA) has emerged as a key focus in medical research due to its clinical significance (1). Circulating tumor DNA (ctDNA), a specific subset of cfDNA, originates from primary tumors and metastatic sites, carrying genomic alterations identical to those found in the primary tumor (2, 3). This makes ctDNA a powerful tool for non-invasive, real-time analysis of tumor dynamics, enabling the monitoring of therapeutic responses, clonal evolution, and resistance development (4).

The detection of ctDNA, however, is often challenging due to its low abundance, as it is heavily diluted by non-tumor cfDNA (5). Despite advancements in detection technologies, sequencing accuracy can be compromised by biological noise, including somatic mosaicism (6). CtDNA is typically extracted from peripheral blood, and its reliability as a biomarker depends on efficient isolation and analytical techniques, which are crucial for consistent quantification and normalization.

Nasopharyngeal carcinoma (NPC), a squamous cell carcinoma originating from the nasopharyngeal cavity’s roof and lateral walls, shows a highly uneven global distribution. More than 70% of new cases cluster in East and Southeast Asia. In endemic regions, over 95% of NPC patients present with non-keratinizing squamous cell carcinoma, strongly associated with Epstein-Barr virus (EBV) (7, 8). The etiopathogenesis of NPC remains incompletely understood, involving EBV infection, environmental factors, ethnic susceptibility, and genetic predisposition. Characterized by an insidious onset, approximately 70% of patients are diagnosed at mid - to - late stages, resulting in a 5-year survival rate of less than 10% (9). With the increasing application of peripheral blood tumor DNA detection, plasma EBV DNA testing has emerged as a valuable tool for NPC diagnosis, prognosis assessment, and minimal residual disease monitoring (8). As ctDNA technology advances, it is anticipated to become a standard approach in comprehensive NPC management.

**Figure 1:**
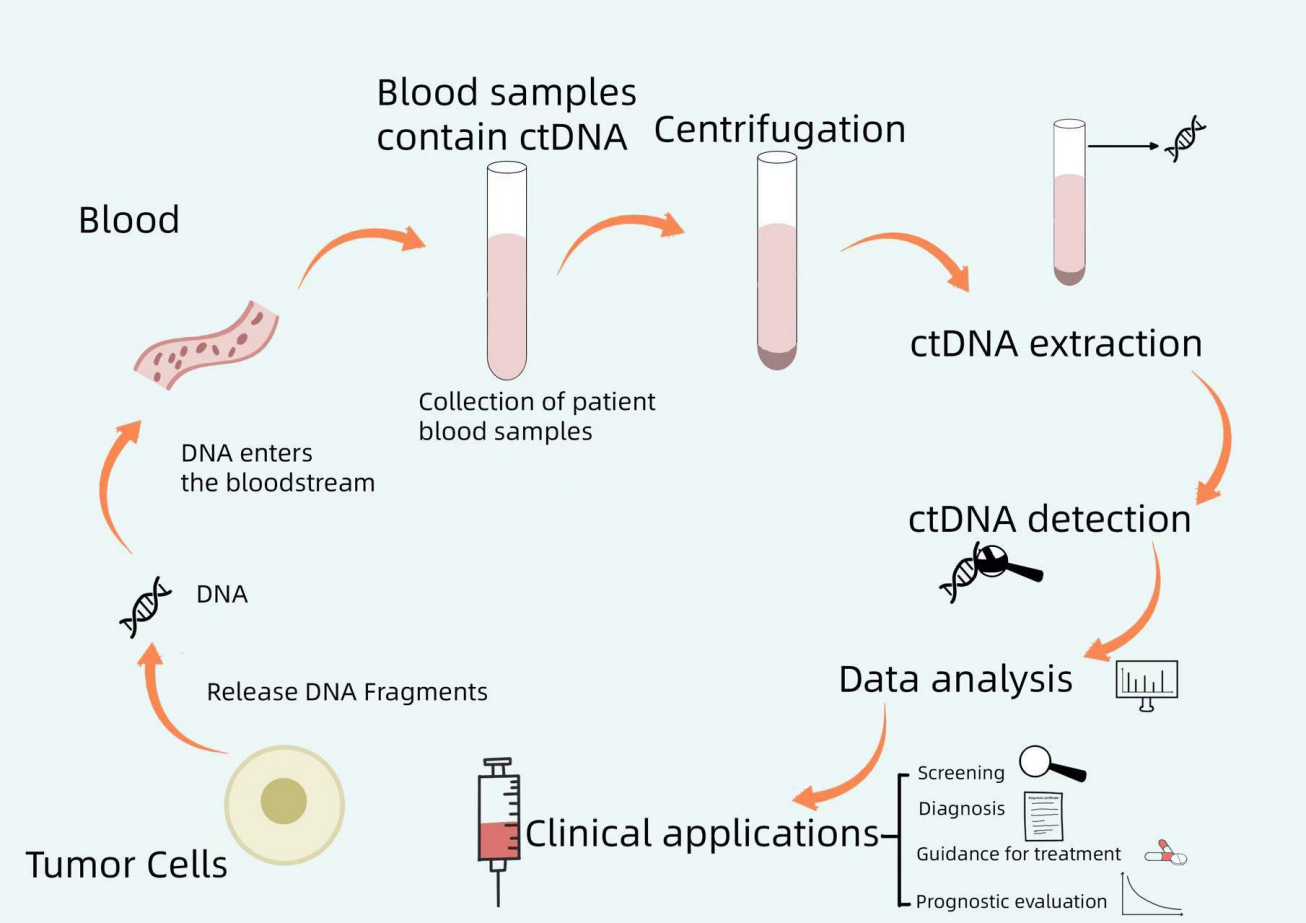
A brief workflow for ctDNA analysis. Tumor cells release DNA fragments into the bloodstream, and circulating tumor DNA (ctDNA) may carry genomic alterations identical to those of the primary tumor. By collecting patient blood samples, centrifuging them, extracting ctDNA from the blood, and performing ctDNA detection, the resulting data can be analyzed to guide clinical screening, diagnosis, treatment, and prognosis assessment.

This review explores the critical requirements for optimal ctDNA analysis and discusses recent advancements in high-throughput and ultrasensitive detection methods. Additionally, it highlights the potential clinical applications of liquid biopsy technologies, with a particular focus on NPC. **Figure 1** shows a brief workflow for ctDNA analysis.

## 2 Pre-analytical considerations for ctDNA

Pre-analytical variables include all steps preceding the analysis of ctDNA specimens and play a critical role in determining ctDNA integrity, purity, and yield, as well as its suitability for subsequent analyses. Despite their importance, these factors are often overlooked during validation, potentially undermining the reliability of results. Establishing standardized pre-analytical protocols is essential to ensure consistency and accuracy in ctDNA analysis (10).

### 2.1 Sample types

Plasma and serum are the most commonly used sample types for ctDNA analysis. However, cfDNA concentrations are reported to be 1–8 times higher in serum compared to plasma due to leukocyte lysis during coagulation and fibrinolysis (11). Consequently, plasma is preferred for ctDNA analysis as it enhances sensitivity and promotes data consistency.

### 2.2 Collection tubes

For ctDNA collection, ethylene-diaminetetraacetic acid (EDTA) tubes are favored over heparin or citrate tubes because EDTA inhibits plasma deoxyribonuclease activity, preserving ctDNA stability (11, 12). However, genomic DNA contamination from leukocytes can occur within four hours of collection if samples are not processed promptly. To mitigate this issue, specialized blood collection tubes (BCTs) with stabilizing agents—such as Streck, Roche, Norgen, PAXgene, and CellSave—have been developed. These tubes extend ctDNA stability, allowing preservation for up to 48 h or longer, which facilitates delayed processing and transportation. While some researchers suggest that proper processing within a few days reduces the importance of specific tube selection, these BCTs remain valuable for scenarios requiring extended sample handling times (13). Specialized BCTs broaden the scope of ctDNA collection and enable delayed transport between clinical center. However, further research is needed to identify subtle differences among these tubes and to establish standardized protocols for optimal ctDNA collection and measurement.

### 2.3 Centrifugation protocols

Efficient ctDNA analysis requires removing heterogeneous content from plasma to ensure the isolation of high-quality ctDNA. Sherwood et al. evaluated single versus dual centrifugation in blood samples from NSCLC patients, finding no significant difference in DNA yield when plasma was centrifuged twice within 2 h compared to a single centrifugation. However, after 72 h, dual centrifugation yielded less DNA, highlighting the influence of protocol timing on DNA recovery (14). Most studies recommend a two-step centrifugation process to optimize cfDNA quality. The initial low-speed centrifugation (800–1,900 g for 10 min) pelts blood cells, followed by high-speed centrifugation (14,000–16,000 g for 10 min) to eliminate remaining cellular debris and improve cfDNA purity (15, 16). Protocols employing extended centrifugation times, such as the adapted (1,900 g for 10 min; 16,000 g for 10 min, at room temperature) and original CEN protocols (1,900 g for 10 min;16,000 g for 10 min, at 4°C), minimize contamination with long DNA fragments compared to shorter centrifugation durations. The adapted CEN protocol may be particularly suitable for ctDNA analysis using cell stabilizer tubes (17). For quality control, plasma should be divided into small aliquots following centrifugation, tailored to specific analytical requirements (18).

### 2.4 Storage conditions

The time and temperature of blood storage before plasma preparation vary based on tube type. Blood in standard EDTA tubes can be stored at 4°C for up to 2 days to reduce cell lysis (12). In contrast, cell stabilizer tubes permit storage at 10°C to 30°C for up to 5 days. Once plasma is separated, freezing at -80°C preserves cfDNA levels for up to 2 weeks, even if the second centrifugation is delayed (19). Although a single freeze-thaw cycle has minimal impact on ctDNA integrity, more than three cycles can degrade nucleic acids, reducing detection efficiency. Long-term storage requirements depend on the intended analysis. Samples stored at -20°C or -80°C for up to 9 months are suitable for mutation detection, whereas ctDNA quantification and fragmentation are optimal within 3 months at -20°C (20). Currently, there is no universal consensus on storage temperatures or durations, emphasizing the need for further standardization.

## 3 ctDNA extraction techniques

Efficient extraction of ctDNA with high yield and purity is critical to ensuring the sensitivity and reliability of downstream analyses. Current DNA extraction methods can be categorized into three main approaches: phase isolation, silica membrane-based spin columns, and magnetic bead-based isolation (21). Silica-based methods leverage the high affinity between the negatively charged DNA backbone and positively charged silica, enabling effective DNA binding. Although phase isolation can achieve high purity, it is more complex and time-consuming compared to other methods (22). Spin column and magnetic bead-based isolation differ primarily in how DNA is captured: in spin columns, DNA binds to a resin, while magnetic beads use a silica-coated surface. Magnetic bead-based systems are particularly efficient at recovering smaller DNA fragments, offering advantages such as lower cost, shorter processing times, and full automation. In contrast, spin column methods are better suited for recovering variable-sized DNA, particularly high molecular weight fragments (>600 bp), and are widely regarded as the preferred choice for general ctDNA isolation due to their reliability and high recovery rates (23). Commercial extraction kits typically employ either spin column or magnetic bead-based approaches. However, novel methods, such as magnetic ionic liquid (MIL)-based extraction, have demonstrated superior performance. For instance, MIL-based dispersive liquid-liquid microextraction (DLLME) combined with direct-multiplex-qPCR enables the simultaneous enrichment of multiple DNA fragments from human plasma with significantly higher enrichment factors than conventional silica-based or magnetic bead methods. This approach holds significant potential for ctDNA detection (24).

Recent advancements in nanotechnology have introduced ultrasensitive magnetic nanowire networks for cfDNA isolation. These structures, characterized by elongated or tubular morphologies and high saturation magnetization, facilitate the efficient capture of cfDNA while minimize loss and degradation, producing high-quality DNA in sufficient quantities (25).

Microfluidic devices for DNA isolation are also under development, classified into solid-phase and liquid-phase isolation techniques. Solid-phase methods employ functionalized surfaces or immobilized beads to capture DNA, while liquid-phase methods utilize chemical reagents or rely on electrophoresis (EP) or dielectrophoresis (DEP) to selectively migrate negatively charged DNA (26). Advances in microfluidic technologies have led to integrated and automated chips and discs capable of isolating ctDNA with high yield and specificity. These devices require minimal sample volumes, reduce processing time, and minimize DNA degradation. They also enhance sensitivity, allowing for accurate quantification and high-throughput screening, making them increasingly feasible for routine clinical applications (27–29).

## 4 Methods for ctDNA detection

ctDNA detection methods can be broadly divided into targeted and untargeted approaches. Targeted methods focus on detecting specific molecular alterations in predefined genes, while untargeted methods extend the genomic scope to identify novel tumor-related alterations, providing potential avenues for advancing cancer therapy (30). Although untargeted methods exhibit high sensitivity, their high cost, long turnaround times, and impracticality for routine clinical use limit their widespread application. **Table 1** compares the advantages and disadvantages of some common ctDNA detection methods.

**Table 1:** Comparison of ctDNA detection methods.

Technique	Approach	Method	Advantage	Disadvantage	Reference
PCR-based	Targeted approaches	Allele-specific PCR	High specificity, economical and easily accessible.	Limited sensitivity, unknown mutations cannot be detected.	(31–33)
		Methylation- based PCR	High specificity, high sensitive.	Sulfite conversion methods result in a loss of DNA information, limited by methylation mutations.	(35, 36)
		Multiplexed targeted PCR	High specificity, high sensitive, multiple mutation detection.	Unknown mutations cannot be detected, cost may increase with the number of targets.	(40–43)
		Digital PCR	High specificity, high sensitive, multiple mutation detection.	Complex operation, expensive.	(44–47)
	Untargeted approach	Enhanced-ice- COLD-PCR	Non-targeted, simple operation, rapid.	Limited quantitative accuracy.	(53)
NGS-based	Targeted approaches	Amplicon−based	High specificity, high sensitive.	Amplification bias, limited detection range.	(55–59)
		Hybrid capture-based	High specificity, high sensitive.	Uneven capture efficiency, complex operation.	(60–63)
	Untargeted approach	Genome-wide analysis	Complete coverage, discovery of new variation.	Complex operation, expensive, complex data analysis.	(66–68)

### 4.1 PCR-based methods

PCR-based methods are the most commonly used techniques for ctDNA detection, offering exceptional sensitivity.

#### 4.1.1 Targeted PCR methods

Targeted PCR techniques employ biological, physical, or chemical methods—such as specific primers, probes, endonucleases, optimized denaturation temperatures, magnetic beads, barcodes, Raman spectroscopy, chemical modifications, and microfluidic chips—to selectively amplify wild-type or mutant sequences. Key approaches in targeted PCR include allele-specific PCR, multi-target PCR, methylation-specific PCR, and digital PCR.

##### 4.1.1.1 Allele-specific PCR

Allele-specific PCR, also known as the Amplification Refractory Mutation System (ARMS-PCR) or PCR amplification of specific alleles (PASA), has been used for detecting hotspot mutations and single nucleotide polymorphisms (SNPs) for years (31). This method employs primers designed to precisely complement the mutation site. DNA polymerase selectively amplifies mutant DNA when the primer’s 3′-end matches the variant base, ensuring high specificity.

The enhanced version, Super-ARMS, further improves specificity and sensitivity through optimized primer design, making it especially suitable for liquid biopsies in NSCLC. Super-ARMS is increasingly used to detect EGFR mutations in plasma (32), with commercially available kits such as the Cobas EGFR Mutation Test v2 and the Super-ARMS EGFR Mutation Test Kit approved for clinical use. These techniques are valuable for detecting T790M resistance mutations during follow-up in NSCLC patients (33). Although ARMS-PCR is cost-effective and widely accessible, its analytical sensitivity and genetic loci are limited. Low ctDNA concentrations and undetected mutations can hinder its broader clinical utility.

##### 4.1.1.2 Methylation-specific PCR

DNA methylation is a key driver of tumorigenesis and tumor progression, making it a valuable biomarker for cancer detection (34). Most ctDNA methylation studies currently rely on bisulfite conversion-based methods, such as methylation-specific PCR (MSP) and Methylated CpG Tandem Amplification and Sequencing (MCTA-seq). For example, Nesvet et al. introduced a method that combines MSP with melt curve analysis using a giant magnetoresistance (GMR) biosensor. This approach enhances methylation detection by employing GMR sensors functionalized with synthetic DNA probes targeting methylated or unmethylated CpG sites. The probes detect melting temperature differences (ΔTm) in MSP amplicons, achieving a detection limit as low as 0.1% methylated DNA in solution. The assay’s multiplexing capability and high sensitivity, without the need for deep sequencing, represent a significant step toward early cancer detection through plasma-based methylation analysis (35). Despite its utility, bisulfite treatment degrades DNA, resulting in the loss of critical methylation data and low-complexity sequencing libraries (36). To overcome these limitations, bisulfite-free enrichment methods have been developed without cytosine conversion, which improve specificity by targeting methylated DNA with anti-methylcytosine antibodies or methyl-CpG binding proteins (37). Aberg et al. demonstrated that optimized Methyl-CpG-binding domain sequencing (MBD-seq) offers distinct advantages for methylome-wide association studies (MWAS). This method provides sensitivity and specificity comparable to whole-genome bisulfite sequencing, even with low-input DNA, while detects a higher density of CpG sites and the largest proportion of CpG islands (CGIs). In the context of limited understanding of methylomes in common diseases, MBD-seq is a valuable tool for identifying disease-associated methylation patterns (38).

##### 4.1.1.3 Multiplex targeted PCR

Multiplex PCR enables the simultaneous amplification of multiple targets in a single reaction. Low-temperature co-amplification (COLD)-PCR is a specialized technique designed to enrich low-abundance mutant sequences amidst wild-type sequences by leveraging critical denaturation temperatures (39). When combined with high-resolution melting (HRM) analysis, Full-COLD PCR offers high sensitivity, simplicity, and cost-effectiveness, making it a promising tool for early-stage breast cancer screening (40). Differential Strand Separation at Critical Temperature (DISSECT) is another effective method for enriching low-frequency mutations. It relies on thermal denaturation of DNA heteroduplexes, eliminating the need for enzymatic reactions. DISSECT shows great potential for routine genetic screening, particularly in cancer detection, and is effective for identifying mutations such as EGFR-resistant mutations and KRAS mutations. Using post-DISSECT Sanger sequencing, KRAS mutations with initial abundances as low as 0.05%–0.1% can be directly detected (41). To address the limitation of targeting a restricted number of mutation sites, advanced techniques such as Simple Multiplexed PCR (SiMSenSeq) and Massively Multiplexed PCR (mmPCR) have been developed. These methods enable the simultaneous detection of multiple mutations. Notably, mmPCR coupled with next-generation sequencing (mmPCR-NGS) can accurately identify copy number variants (CNVs) with mean allele imbalances as low as 0.5%. This approach holds significant promise for diagnosing, characterizing, and monitoring CNV-rich cancers, including breast, ovarian, and lung cancers (42, 43). However, its clinical application remains constrained by the need for prior variant information and the increased costs associated with expanding detection targets.

##### 4.1.1.4 Digital PCR

dPCR partitions DNA into individual reaction compartments, converting the exponential analog signal of conventional PCR into a linear digital signal. This allows for absolute nucleic acid quantification and improves mutation detection by enhancing amplification specificity and minimizing errors. High-sensitivity dPCR methods, such as BEAMing, droplet digital PCR (ddPCR), and Integrated Fluidic Circuit-PCR, have been developed to detect genomic alterations with limits of detection (LoD) as low as 0.01%–0.001% (44, 45). Yin et al. developed a self-priming multiplex dPCR chip capable of detecting four targets using a single fluorescence signal and performing on-chip amplification. This innovation reduced detection time while maintained high accuracy (46). Similarly, Geng et al. designed the integrated droplet digital PCR (IddPCR) microdevice using a “3D extensible” approach. The device addressed challenges in liquid handling, including scaling down from milliliter samples to nanoliter droplets, automating the liquid biopsy workflow, and detecting rare tumor mutations. These advancements hold significant potential for clinical applications (47). However, the widespread clinical adoption of these methods remains limited by their complexity and high cost.

##### 4.1.1.5 Other targeted PCR methods

In addition to PCR-based methods, several alternative approaches have been developed for detecting ctDNA. One such method is the ultra-sensitive assay using mass spectrometry (MS), particularly matrix-assisted laser desorption/ionization time-of-flight (MALDI-TOF). This technique detects multiple mutations with mutant allele fractions (MAF) as low as 0.1% by analyzing the distinct masses of extension products on chips, generating spectrum profiles. The MassARRAY platform has been recognized as a cost-effective tool for multigene profiling, offering reasonable sensitivity and minimal background noise for monitoring tumor burden and genomic changes (48).

Electrochemical biosensors have also shown promise due to their ease of fabrication, portability, low cost, and compatibility with microfabrication and semiconductor technologies. These features enable the rapid development of platforms for ctDNA analysis (49). Key components of these biosensors include bioreceptor selection, bioassay design, and amplification strategies for detecting tumor-specific mutations and methylation events. For instance, Wang et al. developed a label-free electrochemical biosensor incorporating THMS, RNase HII, and TdT dual-enzyme-assisted amplification for ultrasensitive detection of KRAS G12D mutations. By modifying the recognition probe’s loop sequence, this system can be adapted for broader ctDNA detection. This approach holds significant potential for noninvasive liquid biopsy applications (50).

Nanoplasmonic sensing technologies have also garnered attention. Commercial plasmonic sensors are categorized into surface plasmon resonance (SPR), localized surface plasmon resonance (LSPR), and surface-enhanced Raman scattering (SERS). These sensors measure local refractive index changes within small sensor volumes, generating spectral shifts and detecting target molecules with high sensitivity, making them valuable for ctDNA detection (51). For example, a high-throughput SERS-LFA biosensor employing a CHA signal amplification strategy demonstrated ultrasensitive ctDNA detection, proving effective for identifying ctDNA biomarkers (52). However, significant challenges must be addressed before these technologies can achieve widespread clinical application. Efforts should focus on improving their stability and reliability while advance clinical validation and standardization processes.

#### 4.1.2 Untargeted PCR methods

Enhanced-ice-COLD-PCR (E-ice-COLD-PCR) is an untargeted method for detecting all mutations within a defined region of interest. This technique employs chemically modified oligonucleotides to selectively suppress wild-type (WT) sequence amplification, combined with pyrosequencing for mutation detection. Its key advantages include simplicity, ease of assay optimization, compatibility with standard laboratory equipment, and rapid results. These features make it valuable for both basic research and clinical applications, such as identifying clinically significant mutational subclones and tracking therapeutic responses or disease recurrence. However, its inability to deliver highly precise quantification limits its broader use (53).

### 4.2 Next-generation sequencing

PCR-based methods often face challenges such as sequence-specific amplification bias, limited throughput, and slower processing speeds. High-throughput NGS has addressed these limitations, providing a transformative approach to analyzing ctDNA. NGS enables the simultaneous detection of diverse genetic alterations, including single nucleotide variants (SNVs), insertions and deletions (indels), copy number alterations (CNAs), chromosomal rearrangements, and microalterations. The NGS workflow involves four essential steps: library preparation, amplification, sequencing, and bioinformatic analysis. Efforts to enhance ctDNA detection focus on increasing sequencing depth and employing advanced error-correction techniques (54). NGS methods can be classified into two categories: targeted sequencing, which focuses on specific genomic regions, and untargeted sequencing, which provides a broader analysis of genetic alterations.

#### 4.2.1 Targeted sequencing

Targeted sequencing is classified into two main approaches: targeted amplicon sequencing and target hybrid capture sequencing, differentiated by their enrichment strategies. These methods prioritize clinically relevant genomic regions, offering deeper coverage and simplified data processing compared to whole-genome sequencing (WGS) (54).

In targeted amplicon sequencing, notable techniques include Tagged-Amplicon Deep Sequencing (TAm-Seq), the Safe-Sequencing System (Safe-SeqS), and Duplex Unique Molecular Identifiers (UMIs). TAm-Seq utilizes a two-step amplification process to detect mutations with a MAF as low as ~2%, without requiring prior knowledge of tumor-specific alterations. However, its sensitivity is lower than methods such as BEAMing or Intplex (55). Enhanced TAm-Seq (eTAm-Seq™) further improves sensitivity, detecting MAFs as low as 0.25%, and can also identify CNVs, SNVs), and short insertions and deletions (indels) (56). Safe-SeqS incorporates unique identifiers (UIDs) during amplification to reduce NGS artifacts, while methods like Duplex UMI and Cypher-Seq use double-stranded barcoding to minimize errors during library preparation and sequencing (57–59). A significant challenge for NGS-based ctDNA analysis is the lack of robust reference standards for benchmarking performance (59).

Hybrid capture-based methods, such as Cancer Personalized Profiling by Deep Sequencing (CAPP-Seq), provide ultrasensitive detection of SNVs, indels, CNVs, and rearrangements, achieving MAFs as low as ~0.02%. Integrating error-correction systems with hybrid-capture techniques enhances the sensitivity and specificity of ctDNA sequencing (60), enabling the detection of minimal residual disease. Tjensvoll et al. introduced HYTEC-seq, a hybridization- and label-based error correction system that combines molecular labeling and advanced error correction on the Ion Torrent platform. This method, coupled with Plasma Mutation Detector 2, effectively eliminates background noise, allowing highly sensitive ctDNA detection (61). MSK-IMPACT (Memorial Sloan Kettering-Integrated Mutation Profiling of Actionable Cancer Targets), an FDA-approved NGS panel, targets all exons and selected introns of 341 key cancer-related genes. It detects SNVs, indels, CNVs, structural rearrangements, microsatellite instability (MSI), and whole-genome doubling (WGD) (62, 63). In many cancer patients, ctDNA levels often fall below the detection threshold of conventional sequencing methods, especially after treatment (64). Lowering detection thresholds is critical for the broader clinical application of ctDNA technologies.

Broader sequencing approaches, such as whole-exome sequencing (WES), support the discovery of novel driver mutations and therapeutic targets beyond commonly mutated regions. These methods hold promise for cancer screening, diagnosis, prognosis, and treatment. WES, which focuses on coding regions, provides a more streamlined alternative to WGS (65). However, both WES and WGS require substantial DNA input and exhibit limited sensitivity, which diminishes their utility for early cancer detection due to the low background levels of ctDNA.

#### 4.2.2 Untargeted sequencing

Advances in genome-wide analysis have significantly enhanced the detection of ctDNA. For example, AccuScan, a cfDNA WGS technology, achieves single-read genome-wide error correction with an error rate of 4.2×10^-7^, approximately 100 times lower than traditional read-centric de-noising methods. This high-precision approach enables the detection of molecular residual disease with ctDNA sensitivity in the parts-per-million range (66). Digital karyotyping, leveraging high-throughput WGS data, identifies CNVs, while modified rapid aneuploidy screening tests (mFast-SeqS) calculate genome-wide aneuploidy scores. These scores are valuable for stratifying clinical research participants based on tumor burden (67, 68). However, these methods are often cost-prohibitive, technically complex, and involve challenging data analysis due to the high volume and complexity of sequencing output.

### 4.3 Other methods

Single-molecule sequencing, known for its long, accurate reads, provides a scalable and flexible platform for real-time sequencing (69). Prominent technologies in this domain include cSMART and INC-Seq. Hybrid approaches that combine multiple detection methodologies have also emerged. For instance, integrating second-generation ctDNA sequencing panels with microdroplet digital polymerase chain reaction (PCR) and mass spectrometry enables dynamic monitoring of ctDNA. This combined approach effectively captures complex, longitudinal tumor evolution patterns (70). In short, for ctDNA detection technologies to move from the laboratory to clinical application, multiple challenges must be overcome, including technical standardization, cost reduction, data analysis, and clinical validation. By standardizing procedures, reducing costs, enhancing data analysis, and conducting clinical validation, these technologies have the potential to play a more significant role in clinical practice.

## 5 Clinical applications of ctDNA in NPC

Most NPC, strongly linked to EBV, are prevalent in southern China and Southeast Asia. Plasma EBV DNA, a widely used ctDNA marker, shares key molecular features with ctDNA, making it an excellent model for studying ctDNA biology (71, 72). It is pivotal in NPC screening, detection, risk stratification, treatment monitoring, and prognosis evaluation.

### 5.1 Diagnostic applications

EBV DNA is a highly specific diagnostic marker for NPC, with a specificity of 0.96 when compared to other markers such as EA-IgA, VCA-IgA, EBNA1-IgA, and Rta-IgG. Its positive likelihood ratio (PLR) exceeds 10, providing strong evidence for diagnosis. Additionally, EBV DNA demonstrates the highest diagnostic accuracy, with an area under the curve (AUC) of 0.96 (P < 0.05) (73). Target capture sequencing has identified significant differences in the abundance and size distribution of plasma EBV DNA between NPC and non-NPC individuals. These findings have informed the development of a second-generation NPC screening method, which improves diagnostic performance. This approach enables single-point testing without the need for follow-up blood samples, greatly simplifying screening and facilitating large-scale population-level implementation (74). Early detection enhances treatment outcomes, and widespread screening in endemic regions could lead to earlier diagnoses, reduced mortality, and improved patient quality of life. Despite its promise, EBV DNA testing has limitations. Low-volume NPC cases may evade detection, resulting in false negatives. Therefore, its use as a screening tool, even in endemic areas, requires caution. Patients missed during screening might not receive the staging or treatment benefits afforded to those testing positive (75). Ongoing research is essential to safely exclude NPC in clinical settings. Advances in ctDNA testing technologies and comprehensive clinical trials are critical to improving early detection rates and optimizing outcomes for NPC patients.

### 5.2 Guidance for treatment and prognostic evaluation

ctDNA detection has emerged as a potential valuable tool in managing NPC, significantly enhancing risk stratification and enabling precise evaluation of treatment responses. This technology provides critical information for early clinical intervention and supports the personalization and optimization of NPC therapies (76). For example, the Matched WBC Genome sequencing Independent CtDNA profiling (MaGIC) version 2 accurately predicts chemotherapy sensitivity in NPC patients using a single liquid biopsy collected prior to initiating standardized treatment (77). Additionally, ctDNA sequencing can reproduce tumor tissue exome sequencing, while peripheral blood ctDNA offers a non-invasive alternative for treatment decision-making in patients who cannot or choose not to undergo tissue biopsy (78). Combined assays, such as oral brushing combined with plasma EBV DNA detection, further enhance sensitivity and negative predictive value without compromising specificity in detecting local NPC recurrence (71). A large-scale cohort study by Jiawei Lv et al. used qPCR to track circulating free EBV DNA (cfEBV DNA) in NPC patients throughout treatment. The study demonstrated that dynamic cfEBV DNA changes reflect tumor clone behavior and provide real-time risk assessments, highlighting ctDNA’s potential as a biomarker for therapy guidance and monitoring (79). As ctDNA detection technology advances, it is poised to become a standard tool in comprehensive NPC management.

Beyond NPC, ctDNA detection shows great potential in managing other cancers, particularly non-small cell lung cancer (NSCLC). Commercial ctDNA detection kits are now available for monitoring NSCLC patients (33). In colorectal cancer, advancements in analyzing ctDNA methylation and fragmentomics, combined with classification models, enable highly accurate differentiation of blood samples from colorectal cancer patients and healthy individuals, facilitating early detection (80). Additionally, ctDNA analysis can quantify circulating tumor fraction (TF), serving as a tumor-independent prognostic marker (81). Despite its promise, integrating ctDNA detection into clinical oncology practice presents challenges. These include determining optimal sampling time points, setting variant allele frequency (VAF) thresholds, and addressing other technical and clinical complexities. Innovative clinical trials are essential to expand the scope of plasma ctDNA analysis beyond treatment selection. Nonetheless, ctDNA detection holds significant potential to personalize cancer treatments and improve patient outcomes across multiple cancer types (82).

To better understand the behavior of cancer cells and their responses to drugs, and to more effectively introduce ctDNA testing technology into clinical practice, pre-clinical models are of great significance in facilitating this process. Preclinical models mainly include *in-vivo* models and *in-vitro* models. *In-vitro* models, such as *in-vitro* cell line and organoid banks, have the advantages of easy operation and low cost. Some types can also retain cell characteristics and achieve high-throughput screening. However, these models are insufficient in mimicking the tumor microenvironment, suffer from problems of heterogeneity and insufficient representativeness. *In-vivo* models, such as patient-derived xenograft (PDX), patient-derived organoid (PDO), CTC-derived xenograft (CDX), and zebrafish models, can simulate tumor heterogeneity and be used to study disease progression and construct disease models (83–85). Currently, the development of patient-derived models (such as PDX and PDO) has facilitated the research of liquid biopsy in aspects like the exploration of tumor biological behavior, genomic analysis, and drug testing. Studies have demonstrated that the ctDNA levels in the plasma of PDX models can mirror the tumor burden represented by the tumor volume across diverse cancer types. Moreover, PDX models provide a more straightforward and efficient approach to test potential drug targets unveiled by ctDNA sequencing results and to observe treatment effects. Additionally, the PDO model is utilized for high-throughput drug screening, offering an efficient platform for assessing drug efficacy, particularly for patients receiving neoadjuvant therapy. Analyzing ctDNA within PDX and PDO models aids in the discovery of biomarkers and the monitoring of tumor burden. Significantly, the results of ctDNA research in patients and those from derived models can be mutually explanatory and verifiable. Specifically, patient-derived models can complement the analysis of ctDNA in human blood samples (84, 86). In the case of head and neck cancers, preclinical research can leverage NGS and innovative technologies, coupled with the continuous refinement of *in-vivo* models. This enables the acquisition of genomic and multi-omics profiles, the simulation of the natural tumor microenvironment and its drug response, thus enhancing and validating personalized treatment strategies (83). In the future, integrating different research systems will enable us to deeply understand the mechanisms of cancer development at various levels and accelerate the clinical application of liquid biopsy biomarkers.

## 6 Conclusion

The detection of ctDNA through mutation-based assays depends primarily on factors such as the quantity of tumor-derived DNA molecules in the sample, the diversity and clonality of cancer cell alterations, the ctDNA fraction, and the assay’s analytical sensitivity. Technological advancements have focused on optimizing these parameters (5) and improving pre-analytical workflows to maximize the recovery and quality of ctDNA. Key areas of refinement include specimen type, collection tube selection, centrifugation protocols, storage conditions, and ctDNA extraction methods (10, 18). Currently, ctDNA detection is widely used to analyze tumor biology and supports tumor screening, diagnosis, monitoring, and prognosis assessment. However, it cannot yet replace pathological biopsy, the gold standard for tumor diagnosis. Standardizing blood collection and plasma isolation procedures is a crucial step toward clinical application, alongside establishing regulatory frameworks to validate ctDNA as a biomarker in clinical trials. Ongoing research is needed to develop innovative and effective methods for the comprehensive diagnosis, treatment, and management of tumors.
